# Fostering Change from Within: Influencing Teaching Practices of Departmental Colleagues by Science Faculty with Education Specialties

**DOI:** 10.1371/journal.pone.0150914

**Published:** 2016-03-08

**Authors:** Seth D. Bush, James A. Rudd, Michael T. Stevens, Kimberly D. Tanner, Kathy S. Williams

**Affiliations:** 1 Chemistry and Biochemistry Department, California Polytechnic State University, San Luis Obispo, California, United States of America; 2 Department of Chemistry and Biochemistry, California State University, Los Angeles, California, United States of America; 3 Department of Biology, Utah Valley University, Orem, Utah, United States of America; 4 Department of Biology, San Francisco State University, San Francisco, California, United States of America; 5 Department of Biology, San Diego State University, San Diego, California, United States of America; University of Western Sydney, AUSTRALIA

## Abstract

Globally, calls for the improvement of science education are frequent and fervent. In parallel, the phenomenon of having Science Faculty with Education Specialties (SFES) within science departments appears to have grown in recent decades. In the context of an interview study of a randomized, stratified sample of SFES from across the United States, we discovered that most SFES interviewed (82%) perceived having professional impacts in the realm of improving undergraduate science education, more so than in research in science education or K-12 science education. While SFES reported a rich variety of efforts towards improving undergraduate science education, the most prevalent reported impact by far was influencing the teaching practices of their departmental colleagues. Since college and university science faculty continue to be hired with little to no training in effective science teaching, the seeding of science departments with science education specialists holds promise for fostering change in science education from within biology, chemistry, geoscience, and physics departments.

## Introduction

Interest in improving natural sciences education at the college and university level is widespread world-wide [1–6]. In fact, a teaching certificate for higher education is now required for new faculty in Australia, Netherlands, New Zealand, Norway, and the United Kingdom [7] and teaching-focused academics are on the rise in Canada [8], Switzerland [9], and the United Kingdom [10]. Across the globe, science, technology, engineering, and mathematics (STEM) departments in colleges and universities struggle to recruit and retain students who are interested in STEM fields. Further, women are persistently under-represented in most STEM disciplines [11,12], especially women of color [13]. In the United States, the majority of students intending to major in STEM fields do not actually complete a STEM degree [14], and many undergraduates who leave the sciences, as well as many of those who persist, complain of ineffective science teaching [15].

With increased international attention to the quality of university-level teaching [12,16], multiple public and private agencies (e.g. Howard Hughes Medical Institute Foundation, National Science Foundation, European Research Council) have invested many resources toward the development and implementation of innovative curricula, but changes have been limited both in effect and extent [17,18]. Additionally, there is increased interest in improving undergraduate teaching among STEM faculty through summer institutes (e.g., National Academies of Science, both in the U.S. and abroad) and workshops associated with professional societies (e.g., American Physical Society), however, these approaches reach a limited number of all STEM faculty in higher education, in general [17]. Unfortunately, many STEM faculty continue to be hired with little to no support or training in effective science teaching methods, much less opportunities tailored to their institutional and discipline-specific pedagogical needs [19].

Another approach to providing local support to STEM faculty in improving undergraduate science education could be the inclusion of education specialists among faculty within STEM departments. This phenomenon, known as Science Faculty with Education Specialties (SFES), appears to be widespread and increasing over the last decade [20–22] with potentially similar positions being added internationally [8–10,23,24]. Embedding natural sciences education specialists within science departments has enormous potential to influence K-12 science education, to increase discipline-based research in science education, and potentially to improve undergraduate science education [19,25]. For example, natural sciences faculty who incorporate innovations may successfully disseminate their improvements through dynamic interactions with their colleagues [17]. Even with the great potential for advancing change within their departments or “persuading professors” [26], the relative impacts of SFES in these different arenas of science education have not been systematically investigated and reported. Additionally, prior work on the SFES phenomenon has relied on online survey methodologies, which produce relatively limited descriptions of SFES and their professional efforts [22,27–29].

To investigate the SFES phenomenon in more depth in the United States, and SFES perceptions of their professional impact in science education, we conducted an interview study among a randomized, stratified sample of U.S. SFES across a variety of institution types and science disciplines. Interview participants had previously participated in a national, online survey and self-identified as SFES [22,29]. In the current interview study, we examined SFES perceptions of their identity as an SFES, the origins of their faculty positions, their training in science education, their job satisfaction, and most importantly their perceptions of their professional impacts on science education. In this paper, we present only those interview study findings that relate to professional impacts on undergraduate science education reported by SFES, which were surprisingly extensive, varied, and collaborative with departmental faculty colleagues. Additional findings on identity, origins, training, and satisfaction will be described elsewhere. Findings presented here suggest that the majority of SFES interviewed—across all disciplines and institution types studied—report professional impacts strongly linked to improving undergraduate science education.

## Methods

Previous research on the SFES phenomenon has primarily employed online survey methodologies to investigate large numbers of SFES and to attempt to ensure a diversity of perspectives. Here, the research design was purposefully interview-based, to investigate more deeply the experiences of a subset of SFES and to share these experiences using the language of SFES themselves. Below, we describe our sampling procedures, interview protocol and data collection methods, and our mixed methods approach to data analysis, which included describing qualitative themes that emerged from analysis of the interviews, as well as quantitative analyses of the prevalence of these themes among the entire interview sample.

### Sample

To construct our sample for this national interview study of U.S. SFES, we started with a population of SFES who had participated in a previous online survey (n = 289; [22]). Of these 289 individuals, a total of 166 indicated their willingness to participate in future research on SFES positions involving interviews, and provided contact information. These 166 SFES formed the subject pool for our interview study of U.S. SFES. Any of the SFES who originally volunteered to be interviewed would have without doubt provided interesting insights. However, we randomly selected our participants to minimize our own sampling bias and maximize the diversity of perspectives investigated. Stratified random sampling of the subject pool was based on two primary criteria, 1) institution type [PhD-granting, MS-granting, or primarily undergraduate institutions (PUI)] and 2) strong considerations of leaving their current position (staying, leaving). These criteria were selected because previous studies had identified differences in the SFES phenomenon across institution types [22] and because substantial numbers of SFES in both the California State University system [27,28] and across the United States [22] have been found to be seriously considering leaving their current positions. Using these two criteria, we generated six clusters of SFES. Each subject in each cluster was assigned a random number. Sampling within each cluster was prioritized based on two secondary criteria: disciplinary field (in the order of geoscience, physics, chemistry, and biology) and an individual's random number (from lowest to highest).

We selected six individuals with whom we conducted pilot interviews. Because the pilot interview protocol functioned as expected and yielded valuable data similar to later interviews, we included these pilot interviewees in our final sample. After the pilot interviews were conducted, we invited 44 additional people to participate in our study who were distributed across the six clusters. Of those, 38 responded indicating their willingness to be interviewed (86% response rate). Six back-up individuals were similarly selected to replace the six individuals who did not initially respond, bringing our sample size to 50 individuals (including pilots). During the process, internal checks were conducted to ensure that the random sample was balanced across the six clusters. Participants received a $250 honorarium as compensation upon completion of their interview. This study was approved by California State University Los Angeles Institutional Review Board. Subjects provided written informed consent prior to participation following a procedure approved by the aforementioned institutional review board.

### Data collection

Using the contact information provided, subjects were scheduled for 60-minute telephone interviews that were completed between July and September 2013. To orient each participant to the study, we shared, via email, our research goals, the categories of questions that would frame their interview, the identities of the researchers who would conduct the interview, and assurances of confidentiality and protection of identity. If a research team member had extensive familiarity with a participant, they did not conduct that specific interview, so that participants would not assume that the interviewers had prior knowledge of their professional experiences. The interviews were audio-recorded, conducted by two interviewers, and fully transcribed. Although one interviewer took a more active role and asked the majority of questions, a second interviewer was present to ensure consistency in the interview protocol and to provide a back-up recording. During the interview, participants were addressed using their actual name, but during transcription and analysis, pseudonyms replaced actual names and disciplines, and institution names were redacted to protect the identities of our participants.

Interviews were conducted using a semi-structured interview protocol that included an informational preamble followed by seven main questions centered on: 1) the nature of their current position, 2) their identity or non-identity as an SFES, 3) the motivations for the creation of their current position, 4) their perceptions of their professional impact and influence 5) the effectiveness of their training, 6) their job satisfaction, and 7) their viewpoints regarding the SFES phenomenon in general (see S1 File for entire interview protocol).

### Data analyses

While our interviews produced rich and broad descriptions of the professional experiences of 50 individual SFES, here we present evidence and insights only about SFES perceptions of their professional impact upon their institution. This was specifically probed in all interviews with the question: “*What impact or influence do you feel you have had in your position*?*”* Depending on the participant’s response, follow-up questions were asked to probe their perceptions of impact at the level of their department or their institution more broadly. For the analyses presented here, *impact* was defined as evidence of SFES influencing others professionally *within their department or institution*. While *impact* could be interpreted positively or negatively, most SFES reported positive impact. Importantly, this does not imply that SFES have only positive impact, but rather that investigation of negative impacts will likely require interviews with other stakeholders and not SFES themselves, in future studies. Additionally, impacts reported by participants were all coded as such, without researcher judgment on the extent of influence. By accessing the voices of SFES in the results section, readers can evaluate relative degrees of impact. Finally, while much of the evidence about SFES perceptions of impact came from a specific section of the interview, the entire transcript for each participant was analyzed and some evidence about professional impact arose during responses to other interview questions.

Transcripts were analyzed using a grounded theory approach to detect emergent themes across the diverse group of SFES interviewed and through a constant comparative method [30–33]. At least two researchers examined all of the interview responses for each open-ended question, determined emergent themes independently, and then discussed their proposed coding schemes together. Through discussion, common themes that emerged from interview analysis were identified and solidified, often with multiple revisions of the language used to describe the overall theme. Additionally, themes uniquely identified by only one researcher were further discussed, with some eventually being included in common themes and others established as new themes. A revised set of coding themes were then independently used by multiple researchers to re-code interview evidence. This process was iteratively continued until independent coding by multiple researchers for all the evidence resulted in high levels of agreement.

Results from the interviews are reported primarily as descriptive statistics with some parametric comparisons among SFES from different institution types or among types of impacts reported. Differences are not statistically significant unless explicitly stated. When appropriate, Pearson’s χ^2^ tests [34] were used to statistically compare subpopulations of SFES within our sample, and may or may not generalize to all U.S. SFES. Results are displayed in figures for both the aggregate sample and for individual SFES. Emergent themes are represented in figures, as well as Supplementary Information tables with sample quotes.

Finally, while this semi-structured interview dataset from a randomized, stratified SFES sample is rich, varied, and detailed, it is important to remember that data reported here reflect SFES perceptions of their own professional impacts. Future studies would be key in triangulating SFES views with the perceptions of their local faculty colleagues and administrators. Additionally, it important to note that these SFES were those who participated in a national survey of SFES and who then agreed to be interviewed, such that individuals who did not self-identify as SFES are not represented here.

## Results

While science departments appear to have been hiring SFES increasingly over the last decade [22,28], SFES themselves have not been formally queried about their perceptions of the impact of their professional efforts. Below we present results from an interview study with a randomized, stratified sample of SFES (n = 50) across institution types, disciplines, and the United States. Interview results presented here are constrained to only those findings that specifically related to SFES perceptions of impact in the field of science education. First, we present a description of the SFES interview sample, followed by an overview of the proportion of SFES reporting impact in the three arenas of science education: undergraduate science education, research in science education, and K-12 science education. Thereafter, results focus more specifically on the surprisingly extensive impacts reported by SFES in undergraduate science education, for which there were six emergent themes representing different types of impact.

### Description of SFES Interview Sample

**Fig 1:**
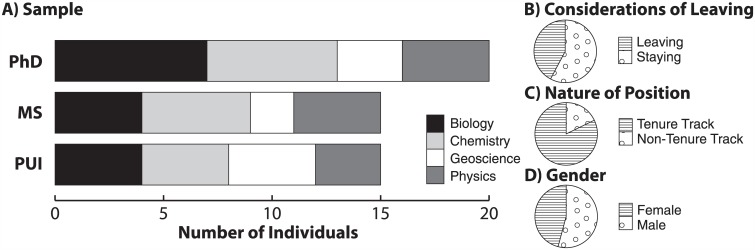
Description of SFES Interview Sample. Reported home institution type (PhD-granting, MS-granting, and Primarily Undergraduate Institutions) disaggregated by science discipline (A), considerations of leaving (B), the nature of the SFES position (C), and SFES gender (D).

**Fig 2:**
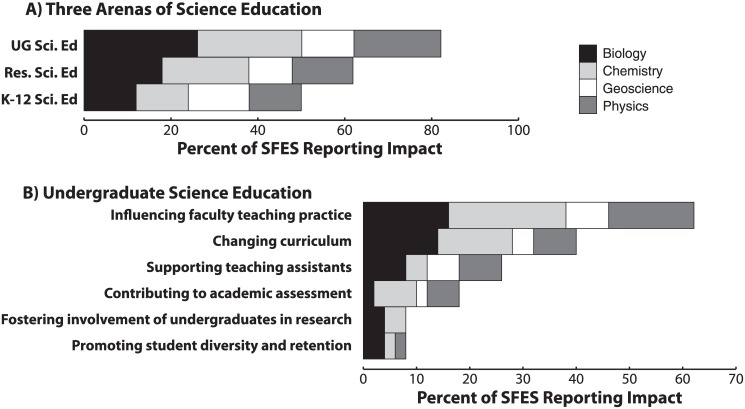
Reported SFES Impact. Proportions of SFES reporting impacts in the three arenas of science education (A) and disaggregated impact themes in undergraduate science education (B).

**Fig 3:**
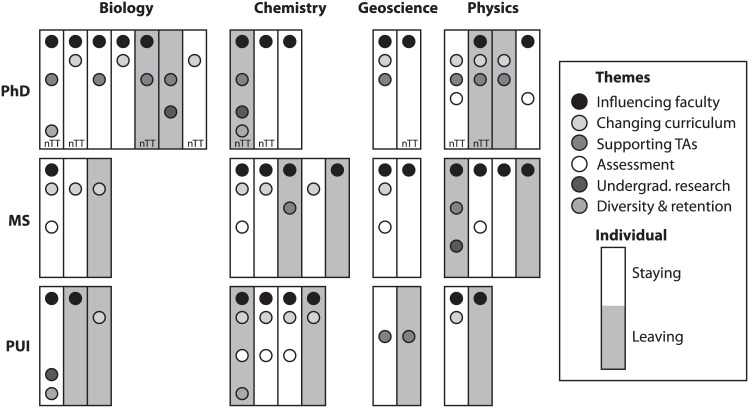
Visual Summary of Individual SFES Impact in Undergraduate Science Education.

The interview sample was generated using two primary criteria: institution type (PhD-granting, MS-granting, or PUI: Primarily Undergraduate Institution) considerations of leaving (staying or leaving) and one secondary criterion: discipline (biology, chemistry, geoscience, or physics). By design, the sample (Fig 1A) included comparable numbers of individuals from PhD-granting institutions (n = 20/50), MS-granting institutions (n = 15/50), and PUIs (n = 15/50). The sample also contained comparable numbers of SFES strongly considering leaving (n = 21) and strongly considering staying (n = 29; Fig 1B). Distribution of subjects across disciplines (Fig 1A) was purposefully relatively similar, and the smallest proportion was affiliated with geoscience departments. The majority of respondents reported being tenured or in tenure-track positions (82%, n = 41/50), with the rest (18%, n = 9/50) filling non-tenure track positions (Fig 1C). Respondents included slightly more males than females (n = 27/50, 23/50, respectively; Fig 1D). Both of these proportions are comparable to SFES distributions seen nationally [22]. Underlying data for Figs 1–3 are available in Tables A-D in S1 File.

### Proportions of SFES Reporting Impact across Three Science Education Arenas

To provide an overall context for SFES impacts in science education, we first quantified the proportion of SFES reporting impacts in the three arenas of science education. Impacts in the arena of undergraduate science education were reported by the most SFES interviewed (82%, n = 41/50; Fig 2A). The second most frequently reported arena of impact by SFES was through research in science education (62%, n = 31/50; Fig 2A). Lastly, impacts in K-12 science education were reported by the fewest SFES interviewed (50%, n = 25/50; Fig 2A). More SFES in our interview study reported impacts in undergraduate science education than in either of the other two arenas (Pearson’s χ^2^ = 11.4, p = 0.003).

Reported impacts in undergraduate science education were further examined by institution type. Given the differences in the missions of these different institution types, one might hypothesize that undergraduate science education impacts might be more prevalent for SFES employed at PUIs than at MS-granting or PhD-granting institutions. However, no significant differences (χ^2^ = 2.12, p = 0.347) were seen between reported impacts in undergraduate science education by SFES at PhD-granting institutions (80%, n = 16/20), MS-granting institutions (93%, n = 14/15), or PUIs (73%, n = 11/15). We revisited the data for the SFES (18%, n = 9/50) who were not coded for impacts in undergraduate science education, and we did not find any evidence of reported impacts in this area. There were no clear patterns across this group of nine SFES. They were distributed across all three institution types and all four disciplines. All nine of these SFES reported impacts in at least one of the other arenas. Finally, there were no significant differences among responses by SFES considering staying or leaving, nor were there differences for SFES in different disciplines.

### SFES Perceptions of Impact in Undergraduate Science Education

From analysis of interview transcripts, six themes emerged that described SFES reported impacts within the arena of undergraduate science education. For these six emergent themes, we present three analyses: 1) a quantitative overview of SFES reporting impact for each emergent theme shown in Fig 2B, 2) representative sample interview quotes, which provide the reader direct access to SFES language used in reporting their impacts in undergraduate science education, and 3) a visual summary of individual SFES impacts shown in Fig 3.

#### Six emergent themes of undergraduate science education impact

Since SFES are most often reporting that their professional impact is in the arena of undergraduate science education, then what more specifically are they doing? To address this, we conducted more detailed qualitative analyses of SFES interview data, revealing six emergent themes of impact in the arena of undergraduate science education (Fig 2B). Below we introduce each of these six emergent themes individually, providing sample evidence from the stories shared by individual SFES. Additionally, we refer the reader to Tables E-J in S1 File, which include additional sample evidence for each emergent theme. In total, Tables E-J in S1 File include sample quotes from 73% (n = 30/41) of the SFES who reported any of the above-mentioned impacts in undergraduate science education. Not all evidence is shown due to space limitations and to protect the anonymity of some participants.

*Influencing faculty teaching practice*: The most prevalent reported impact in undergraduate science education was *Influencing faculty teaching practice* (62%, n = 31/50), which was unexpected. The mechanisms by which SFES reported having this influence varied, but were often associated with: instructional collaborations with other faculty, extended implementation of a particular instructional practice, and/or cultivating faculty interest and conversation in teaching more generally. Below, we explore examples of these mechanisms of influence on faculty teaching practice shared by multiple SFES.

For some SFES, their perceived influence on faculty teaching practices occurred through instructional collaborations that ranged from informal co-planning to more formal co-teaching. Some of these instructional collaborations were initiated by science faculty eager to improve their courses, as described below by George, an SFES at a PhD-granting institution:

“Some faculty in my department actually asked if I was in the department, how come I couldn’t help teach some of their large sections to improve them… In addition, some of the upper level faculty are now coming back to help teach the intro courses, so they can see what we are doing. All of this would be helpful for the vertical alignment, but also if there are techniques we are using in the introductory classes, they can see how we do them, become comfortable with them, and then potentially adopt them for their own.”–George, PhD SFES

Other examples of instructional collaborations that influenced faculty teaching practices were initiated by SFES themselves, as described below by Millie, an SFES at a MS-granting institution:

“I was able to bring folks on one at a time. And they either co-taught with me or started using a student learning assisted model, or student (supplementary instruction) model. That started to change folks’ thoughts about teaching and what our role is as instructors. And that’s helped—getting that individual buy-in and kind of building those relationships and building that support.”–Millie, MS SFES

A second way that SFES reported impacting the teaching practices of fellow science faculty was through the extended implementation of specific instructional practices. For example, Willie is an SFES at a PhD-granting institution who described widespread transition towards clicker use, then leading to more problem-based learning and interactive teaching by many faculty:

“We started in our first year lectures and now our second year lectures are totally clicker-driven with intensive problems. They have added recitations to the second year classes in which students are doing problem-based learning. There are people all through the department who have become much more interactive with their students, much less lecture-oriented, much more project-oriented. The impact has really been on the whole culture of authoritarianism, switching from an authoritarian mode and culture to much more nurturing. Now, I’m not saying it’s 100%. But, I mean, the impact has been dramatic. A significant number of faculty teach differently…”–Willie, PhD SFES

Other examples of specific instructional practices reported as influencing the teaching of other faculty included the use of common assessment tools, white-boarding to promote student sharing of predictions and models, and even the physical conversion of teaching spaces, as described by Lee, an SFES at a PhD-granting institution:

“I have had a huge impact to my department, strictly based on the conversion to studio classes. So, the method in which all of our intro courses are delivered, I have strongly influenced that. That change has been embraced by a lot of the senior faculty. Not 100%, but a large portion of them…”–Lee, PhD SFES

Lastly, many SFES reported influencing faculty teaching practices by more generally cultivating faculty interest in changing teaching practices through either informal or informal discussions. Paul, an SFES at a PhD-granting institution, reported fostering regular departmental conversations about teaching:

“I guess on the department level, I’ve been able to have an impact on trying to make conversation around the teaching culture much more explicit. So, right now we are meeting monthly on a committee that is trying to say, ‘How can you actually change the teaching culture within the department?’”–Paul, PhD SFES

Similarly, Roger, an SFES at a MS-granting institution, reported cultivating departmental conversations about teaching based on assessment evidence and student learning gains, which was additionally being considered in tenure and promotion decisions:

“So now in our department, we share pre/post scores in all the lower division courses and all the upper division courses that can be fit to standardized tests, which is probably better than half of them. We share those openly at the end of each semester…And, so we can all look at it and see how we’re doing in terms of student gains…Those of us that are serious about our craft, compare notes, and discuss these things in a community of practice, not a formal one, but an informal one, and we sure do obsess over those scores, which we now include in tenure and promotion decisions.”–Roger, MS SFES

Additional example evidence reported by SFES about their impact on undergraduate science education through *Influencing faculty teaching practice* is included in Table E in S1 File, though not all evidence is shown to protect the anonymity of some respondents.

*Changing curriculum*: The second most prevalent impact in undergraduate science education reported by SFES was *Changing curriculum* (40%, n = 20/50). SFES impacts within this category were distinct from their impacts on faculty teaching practices. SFES stories about influencing faculty teaching were focused on particular individuals collaborating on pedagogical change, namely “who” was involved, whereas SFES stories about curricular change focused on the development or revision of materials, namely “what” was being taught. Curricular changes were often in the context of laboratory courses taught by graduate teaching assistants and in isolation of working with other faculty.

For some SFES, efforts to improve and revise departmental curriculum was perceived as a key mechanism of their impact on undergraduate science education, as described by both Opal, an SFES at an MS-granting institution, and Patty, an SFES at a primarily undergraduate institution:

“We're kind of the ones who push forward some of the curricular changes … they turn to us, figuring that we are at least much better versed in the literature and what has been done, and kind of where might be a logical place to pick up. So, in that respect, I think we're a resource for the department, that's perhaps a little bit different than somebody who is more classically trained and hasn’t looked at education issues. Just like an organic chemist keeps a lot of the NMR spectrometers running. That's kind of their subspecialty … we keep the instruments that one would use in a classroom, kind of going and moving forward.”–Opal, MS SFES

“I think that when you have someone that's interested in curriculum and interested in instruction, I think it then motivates other faculty to take a look at how they are doing things in the classroom…So, we sat down, and we looked at our curriculum, and we've had some impacts on the curriculum for our pre-professionals… And, we've revised our major, to have closer alignment with the standards of our nation.”–Patty, PUI SFES

Multiple SFES reported that impacts related to changing curriculum were made possible through their ability to successfully apply for federal grant awards, and involved many institutional stakeholders. Such examples from Cerra and Ron, SFES from PUIs, as well as Teresa, an SFES from a PhD-granting institution, are shown below:

“Well, we’ve had a large impact across the College of the Sciences. We definitely have, because we’ve done things like a National Science Foundation Course, Curriculum, and Laboratory Improvement grant on critical thinking…For two years, we took cohorts of 8 faculty across the College of the Sciences…that joined us for a week long summer institute to retool their class to teach for critical thinking … So, so that’s a big impact across the campus.”–Cerra, PUI SFES

“We received an NSF grant to create a set of long-term projects, that will span multiple courses … The main goal is to embed students in something that’s really very close to the process of actually doing scientific research, and from the very beginning. So they start these projects and enter biology from sort of the phenomenological point of view. Pick them up again in general physics to create some quantitative models. And, then pick them up again in some advanced biology courses, where they will elaborate them out into models of biological systems. And, so, from a curriculum development point of view, it’s interesting.”–Ron, PUI SFES

“I've gotten a couple of curricular innovation grants for developing new courses. One of them is being taught by one of the faculty professors in the spring. I've not had a chance to teach it, but I developed the course…so I think I've had quite a bit of influence…I talked to people about education and what kinds of things we could and should be doing, that have been backed up by research.”–Teresa, PhD SFES

Finally, SFES impacts on courses and curriculum were sometimes directly tied to improving student success, as described by Charlie, an SFES at a PUI:

“We took a course that has historically about a 50% success rate, and now has about a 75% success rate for first time through. We spent 18 months with three instructional specialists, an instructional designer, a leader of the center for teaching and learning, a multimedia specialist…redesigning it into a blended course … We are now doing it in (other courses) with remarkable success…”–Charlie, PUI SFES

Additional example evidence reported by SFES about their impact on undergraduate science education related to *Changing curriculum* is included in Table F in the S1 File.

*Supporting teaching assistants*: The third most often reported impact in undergraduate science education by SFES was *Supporting teaching assistants* (26%, n = 13/50). These reported impacts centered on introducing teaching assistants to effective pedagogy and supporting their implementation of these techniques, enabling teaching assistants to develop professionally as instructors and positioning them to be agents of change. For many SFES, supporting teaching assistants involved initiating previously unavailable pedagogical training, as described by Rita, an SFES from a MS-granting institution, and Francine, an SFES from a PhD-granting institution:

“I think (I’ve) had an impact, certainly on TA training. That we’ve got TAs who actually get some pedagogical training before we throw them in the classroom…”–Rita, MS SFES

“…we started training the TAs, because a lot of the TAs were taught traditionally, and we have a lot of also international TAs coming from places all over the world. We wanted to make sure we had some kind of fundamental training. So, we did a serious TA training that hadn't been done here in the past…”–Francine, PhD SFES

For some SFES, their impact through supporting teaching assistants grew into formal graduate coursework in effective teaching—as described by Doug—or extended to other parts of their institution—as described by Dina, both of whom are SFES at a PhD-granting institutions:

“I converted all the labs to inquiry labs and trained all the grad students to teach those labs…So, all the grad students are getting exposed to that kind of thinking. Then, I started this grad course, and in its third iteration, it's getting a lot of attention from others…”–Doug, PhD SFES

“We started a TA training program that ended up spawning a whole letters and sciences TA training program…”–Dina, PhD SFES

Finally, Paula, an SFES from a PhD-granting institution, considered her greatest impact on undergraduate science education to be via her work with teaching assistants:

“I think the greatest impact I’ve probably had is with the graduate students within the department. I’ve worked for five years with grad students, teaching a graduate seminar course and I think that has had the greatest impact, in terms of how our teaching is viewed within the department, because these graduate students are kind of the front lines. They’re out there teaching and planning what's going to be taught, and they'll go back and tell their faculty, you know, you should be doing this, you should be doing this, you should be doing this, within your classroom, and then they’ll sort of seek me out and say, What do you do? I'm kind of curious about that, I want to know about that…”–Paula, PhD SFES

Additional example evidence reported by SFES about their impact on undergraduate science education through *Supporting teaching assistants* is included in Table G in S1 File, though not all evidence is shown to protect the anonymity of some respondents.

*Contributing to academic assessment*: The fourth most often reported impact in undergraduate science education by SFES was *Contributing to academic assessment* (20%, n = 10/50). For some SFES, their assessment expertise had an impact primarily at the departmental level, as described by Cerra, an SFES at a PUI, and Wendy, an SFES at an MS-granting institution:

“I think all of us have a big impact on our science departments through assessment…There’s been a big push at our university, and all other universities, for more accountability and for us to have programmatic assessments in place. And, in order to have programmatic assessments, you have to have student learning outcomes for your courses. Our departments would not have gotten very far with that, or it wouldn’t have been very sophisticated assessment, without the science education faculty.”–Cerra, PUI SFES

“We had to develop an assessment plan for our department. Trying to understand what it means to do outcomes assessment and to assess the learning of our students and to sort of set up learning targets and goals and outcomes, even just on that small of scale, that was pretty major… I led that effort and tried to help people understand what that might look like… Our department was charged with doing that, coming up with an outcome assessment document, and nobody really knew what that meant. So, in our department meetings, we hashed that out, and I think because I knew a little bit more about it, I helped lead that.”–Wendy, MS SFES

For other SFES, their assessment expertise had an impact that extended more broadly at their institution, as described by Theodora, an SFES at a PUI, and Lee, an SFES at a PhD-granting institution:

“I was Director of Evaluation and Assessment, and I’ve been a member of the Assessment Subcommittee of the Curriculum Committee for a number of years … So, when I was Director of Evaluation and Assessment that was the year that all the departments were required to write intended learning outcomes for their majors. And they were given guidelines: “Write three to five, and use them in a way that makes them measurable.” And when we had 100% compliance on that, it was just astonishing to me! It’s still astonishing to me to think about. Everybody did this…”–Theodora, PUI SFES

“We were in trouble with our regional accreditor … for not taking assessment seriously … And (the new administration) realized they needed people who had a history at the university who knew what had been going to move this forward. So, there are actually three of us faculty members. I have two colleagues who serve the same role, and they each serve two colleges, themselves …We had all been involved in assessment in our departments or colleges in some way or another, so we kind of took on the lead role for moving things forward, working with the new administration…”–Lee, PhD SFES

Additional example evidence reported by SFES concerning their impact on undergraduate science education through *Contributing to academic assessment* is included in Table H in S1 File.

*Fostering involvement of undergraduates in research and Promoting student diversity and retention*: Two other categories of impact: *Fostering involvement of undergraduates in research* (8%, n = 4/50), and *Promoting student diversity and retention* (8%, n = 4/50) were reported less frequently and are summarized in Tables I and J in S1 File, respectively. All evidence from these two categories is shown in the tables, given the relatively small number of impacts reported in these areas by SFES.

*Differential reporting of themes by SFES from different institution types*: Finally, one might hypothesize that these six emergent themes of impact on undergraduate science education might have been differentially reported among SFES from different institution types. Quantification of specific emergent themes of impact in undergraduate science education showed some differential prevalence among SFES at different institution types. For example, the most frequently reported impact theme was *Influencing faculty teaching practice* (62%, n = 31/50), with more SFES at MS-granting institutions reporting this impact than SFES at other institution types. Perhaps not surprisingly, a greater proportion of SFES at PhD-granting institutions reported the theme *Supporting teaching assistants* than did SFES at other institutions, since PhD-granting institutions may just employ more teaching assistants than do MS-granting or PUI institutions.

#### Visual summary of impacts in undergraduate science education reported by individual SFES

The majority of SFES who asserted that their professional impact was in undergraduate science education reported that this was through *Influencing faculty teaching practice* (62%, n = 31/50). So in that case, are the other emergent themes a result of concentrated efforts among a small number of SFES? To investigate this possibility, we constructed visual representations of reported impact themes for individual SFES. Of those SFES who reported impacts in undergraduate science education, the majority (61%, n = 25/41) reported impacts in at least two of the six emergent themes (Fig 3). In Fig 3, each rectangle represents an individual SFES with individual SFES then grouped by institution type (top to bottom) and discipline (left to right). Shading of rectangles was used to differentiate those who were strongly considering leaving from those who were not. Shaded dots are used to indicate reported impact in each emergent theme. Those marked nTT are individuals who reported occupying a non-tenure-track position at their institution. As an example, the top, left most individual is an SFES at a PhD-granting institution who is in a biology department and is not seriously considering leaving their position. This particular individual reported impacts in three different themes in the arena of undergraduate science education: *Influencing faculty teaching practice*, *Supporting teaching assistants*, and *Promoting student diversity and retention*. Finally, this individual is in a non-tenure-track position.

The patterns across individuals demonstrate that the reported impacts in undergraduate science education are not concentrated in a few SFES, but rather spread across individual SFES from different institution types and disciplines. Further, the profiles of non-tenure-track and tenure-track faculty appear similar both in the number and kind of different undergraduate science education impact themes reported. One exception to the dispersed pattern of impacts was observed for the emergent theme *Contributing to academic assessment*, which was reported by SFES in all disciplines at MS-granting institutions, but was reported only by Physics SFES at PhD-granting institutions (2 out of 20 SFES at PhD institutions) and only by Chemistry SFES at PUIs (3 out of 15 SFES at PUIs). However, this result is drawn from a small population of SFES and may not generalize.

## Discussion

With the pressing global need for improving student experiences in college and university science courses, and the resources being directed to effect change in this area, reform efforts in science education continue to be numerous [3]. A potential, but understudied, reform approach that has been initially described in the United States is the incorporation of SFES into science departments, first investigated in 2006 [35]. Studies done in single states [27,28], regionally [20], and nationwide [22] suggest that the SFES phenomenon is growing. Similar roles are on the rise internationally as well [8–10,23,24]. Previous research has suggested that individual SFES professional efforts often span all three arenas of science education: research in science education, K-12 science education, and undergraduate science education [22,27,28]. A limitation of previous research was its primary use of survey methodology, which provided little opportunity to collect deeper and richer data sets on the SFES phenomenon. Here we discuss implications of SFES perceptions of their own professional impact as shared through semi-structured interviews with a random, stratified sample of SFES. Given the recent interest in discipline-based science education research [6] and the fact that SFES themselves perceived their hiring to be most linked to science teacher preparation [29], one might expect the most prevalently reported SFES impacts to be in one of these two science education arenas. Indeed, more than half of SFES interviewed reported professional impacts either in research in science education (62%) or in K-12 science education (50%). However, the most prevalently reported SFES impacts centered mainly on improving undergraduate science education (82%), which was reported most often as influencing faculty teaching practices. Below we consider the implications of these findings and hypotheses to be tested in future research.

### Questioning assumptions about the SFES model

Our findings refute the notion that SFES are *only* doing discipline-based science education research [6], *only* focusing on K-12 teacher preparation [29], or *only* teaching [10,23,24]. Instead, SFES are often involved in all three arenas of science education (research in science education, K-12 science education, and undergraduate science education). However, more SFES reported impacts in undergraduate science education than impacts in the other areas of their professional work.

Reasons for hiring SFES in science departments have been varied and unclear in previous reports. Some evidence has suggested that SFES are hired to teach and coordinate courses with large enrollments, to save money in times of budget limitations, or to pursue grant money for research in science education [28]. Others have asserted that SFES are hired to focus on program assessment [36] or to collaborate with College of Education faculty [37,38]. Previous research on SFES perceptions of reasons for their hiring suggested that SFES perceive they were hired primarily to prepare future science teachers and to teach certain classes, depending on institution type [29]. Data from this interview study suggest that, regardless of the reasons for their hiring, SFES themselves perceive that most of their impacts are in the area of undergraduate science education. These data are consistent with previous survey findings in which SFES considered being a pedagogical resource for faculty as a key way they could positively influence science education [29] as agents of change [26,39].

One might hypothesize that SFES at PUIs and MS-granting institutions might be more likely to report impacts in undergraduate science education. However, the findings of this study revealed no significant difference among SFES at different institution types (80% PhD, 93% MS, 73% PUI). We found this particularly striking given the widely held view that faculty at PhD-granting institutions focus primarily on research. Additionally, our findings show that 75% of SFES at PhD-granting institutions reported influencing their faculty colleagues’ teaching practices (12 of 16 who reported this impact), which might not be expected from faculty at research-focused universities. Furthermore, while it may be asserted that SFES at PhD-granting institutions are solely engaged in discipline based science education research, this also was not supported by our findings. Given the pressing need for science education reform, the positioning of SFES in science departments across a variety of institution types may promise a local and familiar mechanism for cultivating and driving change.

### Acting as local change agents

*“I’ve been able to have an impact on trying to make conversation around the teaching culture much more explicit*. *So*, *right now we are meeting monthly on a committee that is trying to say*, *‘How can you actually change the teaching culture within the department*?*’”–Paul*, *PhD SFES*

The majority of SFES appeared to have impacts on undergraduate education as local change agents. SFES reported influencing faculty teaching practices through building collaborations, promoting pedagogical communities, and cultivating conversations about teaching, as well as through more expected paths like formally presenting education research or offering professional development (See Table E in S1 File for more examples). Specifically, SFES reported co-teaching together in classrooms, collaboratively planning for class sessions, or strategizing about articulating teaching approaches and content between introductory and upper division courses. These SFES examples of instructional collaborations with departmental faculty would appear to move undergraduate science education reform efforts away from single individual pioneers to a more collaborative model of shared effort, mutual learning, and on-going support [40–42] and are described as “bottom-up” initiatives in an assessment of higher education in 20 countries across the globe [5]. In addition, SFES reported promoting changes in teaching through adoption of clickers and white-boards, as well as physical alterations of classrooms that appeared to encourage faculty implementation, without necessitating direct collaboration with SFES. The effectiveness of extended, long-term interventions like these has been highlighted as a means of facilitating change in undergraduate STEM instructional practices previously [17,43]. Perhaps more generally, many SFES reported that their presence in the department brought a new or renewed focus on teaching to faculty discussions and department meetings, either at the direction of departmental leaders or by faculty themselves. These SFES interactions focused on colleagues may be examples of valuable emerging and informal professional learning communities [39,42,44]. Overall, SFES reported impacts on faculty practice appeared to have relied heavily on use of their human capital, relationship building, and translational expertise, as opposed to direct sharing of educational research evidence on effective teaching. Although we directly probed SFES about their impacts and not about the challenges they faced while trying to make an impact, we were surprised that the SFES interviewed for this study reported minimal resistance to efforts to improve undergraduate science education among the majority of their faculty colleagues.

The interview sample for the study was purposefully constructed to include voices of those SFES who had seriously considered leaving their position, and we anticipated that reported impacts among these SFES would be low. However, there were no apparent differences in reported impacts on undergraduate science education among those SFES considering leaving their position. For example, the quote that began this section was from Paul, an SFES at a PhD-granting institution who had reported seriously considering leaving his position. In short, SFES who were considering leaving their position still highlighted perceptions of impact in undergraduate science education.

### Bringing new skill sets for new challenges

*“*…*just like an organic chemist keeps a lot of the NMR spectrometers running*. *That's kind of their subspecialty* … *we keep the instruments that one would use in a classroom*, *kind of going and moving forward*.*”–Opal*, *MS SFES*

Interviewed SFES reported bringing new sets of skills tied to their educational expertise, which in turn impacted their science departments in ways that went beyond their own teaching and research. Some SFES—like Opal above—described these contributions as being similar in kind to other science colleagues in their departments, but specifically arising from their educational expertise and skills. Of all the SFES interviewed, 62% (31 of 50) described their professional impacts in terms one or more of the following education skill sets: fostering curriculum change (40%), sharing pedagogical skills with teaching assistants (26%), and supporting assessment of student learning (20%). While less than half of SFES reported any one of these as a mechanism of impact, SFES collectively appeared to be bringing these new educational skill sets to undergraduate reform efforts in a variety of ways. The vast majority of SFES who reported impacts in undergraduate science education described bringing at least one of these skills sets to their department (76%; 31 of 41). Those individual SFES who did not report impacts related to these skill sets did not appear to be concentrated in any particular institution type or discipline.

Of the new educational skill sets SFES cited in their professional impacts in undergraduate science education, changing curriculum was most often reported (40%). SFES examples of curricular change were highly varied, spanning science major and non-science major courses, as well as lecture courses and laboratory courses. SFES interviewees commonly discussed revising laboratory curricula, which are generally taught by graduate student teaching assistants or non-permanent instructors, by moving these teaching and learning materials towards a more inquiry-based approach. Interestingly, these curriculum changes were, in some cases, discussed as happening in courses for which the SFES themselves were never the primary instructor, or in courses that they were no longer teaching. This distinction is important as SFES efforts in changing curriculum may foster reforms that could be sustained beyond their direct involvement and that may provide more innovative materials for other instructors. Funding to accomplish curricular reforms was not specifically probed in interviews, but multiple SFES described specific funding mechanisms as key to changing curriculum, often funding from the National Science Foundation. Further, some SFES reported being looked to as curriculum reform experts by their colleagues, a finding that is consistent with the model described by Beach et al. [45] where change agents may teach other individuals about new curricula.

In addition, some SFES (26%; 13/50) reported bringing their educational skills to future scientists through supporting both graduate and undergraduate teaching assistants. Calls for the integration of pedagogical training into the education of future scientists in doctoral programs are uneven at best. Currently, learning how to teach the science they know to others is a choice that scientific trainees themselves make, and it is often considered “extracurricular” to their dissertation or post-doctoral research. SFES interviews revealed a range of approaches to supporting both graduate teaching assistants (GTAs) and undergraduate teaching assistants. For GTAs, some SFES offered pedagogical courses for graduate students such as those described by Schussler et al.[46], or directly mentored GTAs in the context of coordinating laboratory courses [47]. SFES also reported impacting undergraduate teaching assistants, sometimes called learning assistants, who are often undergraduate alumni of courses that return as near-peer mentors for currently enrolled students. While a less prevalently reported influence on undergraduate science education, this role of SFES could provide a systematic way for all college and university science departments to provide training in effective science teaching for future faculty during their graduate (and even undergraduate) school years.

Finally, a subset of SFES interviewed (18%; 9/50) reported bringing skills in assessment of student learning to their departments. Increasingly, colleges and universities of all types are being asked to demonstrate their educational value to external stakeholders, accreditors, and even parents and families through direct assessment of student learning [48]. Not surprisingly, the expertise and skills required to design, collect, and analyze these types of assessment data are often absent from most academic departments, including science departments [49,50]. However, SFES may possess assessment and evaluation skills not shared by basic science colleagues, either from their own teaching efforts or in relation to their own scholarship and discipline-based education research programs. For example, in their study examining factors that promote faculty involvement in and satisfaction with academic program and student assessment, Grunwald and Peterson [51] described the value of collaborative activities like professional development opportunities, distributing evidence of the benefits of student assessment, and promoting faculty interest in teaching and instructional methods—all impacts reported by SFES. Interviewed SFES that reported having an impact on undergraduate science education through assessment shared examples of leading efforts in developing student learning outcomes, designing measurement tools, and fostering regular conversations to review assessment outcomes and implications. While only 18% of SFES interviewed discussed assessment as part of their professional impact, it is important to note that the interviews did not specifically probe SFES about their involvement in departmental assessment, but rather asked them broadly about their professional impact. As such, this may be an underestimate of SFES involvement in departmental assessment efforts. Multiple SFES who had reported that they had impacts through contributing to academic assessment felt that the influence was large and positive, and that their expertise was welcomed and valued by their colleagues.

### Testable Hypotheses and Future Directions

The finding that the highest proportion of SFES perceived impacts on undergraduate science education sets the stage for further questions and hypothesis-testing in this arena. For example, what is the alignment between SFES perceptions of impact and the perception of their departmental colleagues? What is the state of undergraduate science education reform in departments with and without SFES? How do SFES function as agents of change? Are their efforts explicit or more subtle? How do SFES go about cultivating faculty conversations about teaching? Additionally, promoting equity and diversity is a key piece of undergraduate science education reform efforts, yet only four of the 50 SFES interviewees mentioned involvement in equity and diversity efforts. Future research could more directly probe SFES, by asking them to describe their professional efforts in this area. Finally, it is important to note that while impacts on undergraduate science education were most prevalently reported in our interview study, many additional impacts in the arenas of research in science education and K-12 science education were also reported and will be explored in future publications.

### In Conclusion

Improvement of science education is a pressing issue globally [4,11,12,16]. Unfortunately, STEM faculty in higher education continue to be hired with little to no training in effective science teaching. The seeding of college and university science departments with science education experts—Science Faculty with Education Specialties—holds promise in providing local expertise to support improved science teaching and learning. While it is unclear how many college and university science departments currently have an SFES in one or more natural sciences departments, the integration of specialized expertise in science education within departments increasingly appears to be a promising mechanism for driving local reform. This deeper investigation of SFES through interviews with a randomized, stratified sample of U.S. SFES revealed surprisingly extensive, varied, and collaborative impacts upon undergraduate science education, with the majority of SFES reporting influencing departmental faculty colleagues’ teaching practices. This is in alignment with the suggestions of Beach et al. [45], who have recommended that change agents work locally at the department level. Key to this within-department change may be the fact that SFES generally are like other scientists in their departments, being faculty with research agendas of their own who also have skills and knowledge that can support departmental aspirations for instructional improvement and change. From our sample, a majority of SFES was tenure-track faculty, with SFES at PhD-granting institutions being the most likely to occupy non-tenure-track faculty positions. One wonders if the tenure-track nature of the majority of SFES positions was helpful in their ability to influence the teaching practice of their colleagues. Additional research studies are necessary to further explore the SFES phenomenon, including investigations of the perspectives of non-SFES stakeholders.

## Supporting Information

S1 File Supplementary Information File. The Supplementary Information file contains the following information to support readers’ exploration of the study results: 1) Minimal Data Set for Figures (Tables A-D) 2) Quote Tables (Tables E-J), and 3) Interview Protocol.
(PDF)
