# Supplementary material for: Fostering Change from Within: Influencing Teaching Practices of Departmental Colleagues by Science Faculty with Education Specialties
Source: PLoS One. 2016 Mar 8;11(3):e0150914. doi: 10.1371/journal.pone.0150914 (PMC4783031; doi:10.1371/journal.pone.0150914)
Supplement: S1 File — The Supplementary Information file contains the following information to support readers’ exploration of the study results: 1) Minimal Data Set for Figures (Tables A-D) 2) Quote Tables (Tables E-J), and 3) Interview Protocol. (PDF) [file pone.0150914.s001.pdf]

Supplementary Information

Below, we have included three sections of supplementary information to support readers' exploration of the study results: 1) Minimal Data Set for Figures, 2) Quote Tables, and 3) Interview Protocol.

The following abbreviations as used throughout the tables:

PHD = SFES employed at a PhD-granting institution
MS = SFES employed at a MS-granting institution
PUI = SFES employed at a Primarily Undergraduate Institution

1. Minimal Data Set for Figures

Table A: Supporting data for Figure 1A. Sample counts split by institution type and science discipline.

	Biology	Chemistry	Geoscience	Physics	Total
PHD	7	6	3	4	20
MS	4	5	2	4	15
PUI	4	4	4	3	15
Total	15	15	9	11	50

Table B: Supporting data for Figure 1B and 3. Sample counts for considerations of leaving, the nature of the position, and gender, split by institution type and discipline. Figure 1B uses only the summary totals.

		Biology	Chemistry	Geoscience	Physics	Total
Not Seriously Considering Leaving (Stayer)	***PHD***	5	3	2	2	12
	MS	2	3	2	2	9
	PUI	2	2	2	2	8
	Total	9	8	6	6	29
Seriously Considering Leaving (Leaver)	***PHD***	2	3	1	2	8
	MS	2	2	0	2	6
	PUI	2	2	2	1	7
	Total	6	7	3	5	21
Tenure Track	***PHD***	3	4	2	2	11
	MS	4	5	2	4	15
	PUI	4	4	4	3	15
	Total	11	13	8	9	41
Non-Tenure Track	***PHD***	4	2	1	2	9
	MS	0	0	0	0	0
	PUI	0	0	0	0	0
	Total	4	2	1	2	9
Female	***PHD***	3	0	1	3	7
	MS	2	3	1	1	7
	PUI	3	3	2	1	9
	Total	8	6	4	5	23
Male	***PHD***	4	6	2	1	13
	MS	2	2	1	3	8
	PUI	1	1	2	2	6
	Total	7	8	5	6	27

Table C: Supporting data for Figure 2A and 3. Sample counts for reported impact in the Three Arenas split by institution type and discipline. Figure 2A uses only the summary totals for each arena. Figure 3 uses only the counts for the Undergraduate Science Education arena. Additional data, not appearing in Figure 2, are included for completeness.

		Biology	Chemistry	Geoscience	Physics	Total
**Undergraduate Science Education**	***PHD***	7	3	2	4	16
	MS	3	5	2	4	14
	PUI	3	4	2	2	11
	Total	13	12	6	10	41
**Research in Science Education**	***PHD***	4	4	2	2	12
	MS	2	3	0	3	8
	PUI	3	3	3	2	11
	Total	9	10	5	7	31
**K-12 Science Education**	***PHD***	1	1	2	1	5
	MS	4	4	2	3	13
	PUI	1	1	3	2	7
	Total	6	6	7	6	25

Table D: Supporting data for Figure 2B and 3. Sample counts for impact themes in Undergraduate Science Education split by institution type and discipline.

		Biology	Chemistry	Geoscience	Physics	Total
**Influencing faculty teaching Practice**	***PHD***	5	3	2	2	12
	MS	1	4	2	4	11
	PUI	2	4	0	2	8
	Total	8	11	4	8	31
**Changing curriculum**	***PHD***	3	0	1	3	7
	MS	3	3	1	0	7
	PUI	1	4	0	1	6
	Total	7	7	2	4	20
**Supporting teaching assistants**	***PHD***	4	1	1	3	9
	MS	0	1	0	1	2
	PUI	0	0	2	0	2
	Total	4	2	3	4	13
**Contributing to academic assessment**	***PHD***	0	0	0	2	2
	MS	1	1	1	1	4
	PUI	0	3	0	0	3
	Total	1	4	1	3	9
**Fostering involvement of undergraduates in research**	***PHD***	1	1	0	0	2
	MS	0	0	0	0	0
	PUI	1	1	0	0	2
	Total	2	2	0	0	4
**Promoting student diversity and retention**	***PHD***	1	1	0	0	2
	MS	0	0	0	1	1
	PUI	1	0	0	0	1
	Total	2	1	0	1	4

2. Quote Tables

Pseudonyms have been used below to protect the anonymity of participants.

Table E: SFES Impact on Undergraduate Science Education – Influencing Faculty Teaching Practices (n = 31/50; 62%)

Sample evidence
I have had a huge impact to my department, strictly based on the conversion to studio classes. So, the method in which all of our intro courses are delivered, I have strongly influenced that. That change has been embraced by a lot of the senior faculty. Not 100%, but a large portion of them... – Lee, PhD SFES
My chair asked me and no other faculty member in our department of 25, to do a presentation to the rest of the faculty at the opening department meeting...to talk about the state of the [discipline] education program: What we're doing…Who are we graduating? Where are we placing them? What grants are we involved in? …And, I think it was enlightening for people. It generated some conversation, and I think it just helped to positively dispose the faculty towards our positions … - C.K., MS SFES
We started in our first year lectures and now our second year lectures are totally clicker-driven with intensive problems. They have added recitations to the second year classes in which students are doing problem-based learning. There are people all through the department who have become much more interactive with their students, much less lecture-oriented, much more project-oriented. The impact has really been on the whole culture of authoritarianism, switching from an authoritarian mode and culture to much more nurturing. Now, I'm not saying it's 100%. But, I mean, the impact has been dramatic. A significant number of faculty teach differently... – Willie, PhD SFES
Well, I think I have helped increase the quality of education, being administered by my colleagues, because I am able to provide a resource that they wouldn't necessarily have. … I think there is something about my specialty that people acknowledge and value for input. – Carter, MS SFES
Some faculty in my department actually asked if I was in the department, how come I couldn't help teach some of their large sections to improve them…Some of the ideas that I've been trying to incorporate into introductory [discipline], I can help incorporate some of these ideas into the upper level courses and help establish some alternative practices in these other classes as well. In addition, some of the upper level faculty are now coming back to help teach the intro courses, so they can see what we are doing. All of this would be helpful for the vertical alignment, but also if there are techniques we are using in the introductory classes, they can see how we do them, become comfortable with them, and then potentially adopt them for their own. – George, PhD SFES
I guess on the department level, I've been able to have an impact on trying to make conversation around the teaching culture much more explicit. So, right now we are meeting monthly on a committee that is trying to say, "How can you actually change the teaching culture within the department?" – Paul, PhD SFES
There's a very traditional faculty-member… In the past year and a half, he has completely blossomed as someone who is like, "I teach, and I care." He's trying all these new things, and yesterday he put in an education research proposal to reform his class…and that has been just really awesome to watch and participate in. My grad student is taking some of her data in his class, and it is partly because of his interactions with her that he's started down this path … – Allison, PUI SFES
And so, I was able to bring folks on one at a time. And they either co-taught with me or started using a student learning assisted model, or student (supplementary instruction) model. That started to change folks' thoughts about teaching and what our role is as instructors. And that's helped – getting that individual buy-in and kind of building those relationships and building that support. – Millie, MS SFES
So now in our department, we share pre/post scores in all the lower division courses and all the upper division courses that can be fit to standardized tests, which is probably better than half of them. We share those openly at the end of each semester…And, so we can all look at it and see how we're doing in terms of student gains...Those of us that are serious about our craft, compare notes, and discuss these things in a community of practice, not a formal one, but an informal one, and we sure do obsess over those scores, which we now include in tenure and promotion decisions. – Roger, MS SFES

Table F: SFES Impact on Undergraduate Science Education – Changing Curriculum (n = 20/50; 40%)

Sample Evidence
I was able, with the support of the department, to computerize the freshman, non-majors labs. We completely revised the program, so all of our freshmen – both majors and non-majors, are doing open-ended labs, where they actually have to develop a hypothesis. They actually have to develop some real thinking. I developed a series of homework assignments that corresponded with the lecture and the lab that the students would turn in that are graded by the TA, so it's weekly graded. The questions require much more in-depth thinking… – Uma, MS SFES
And, so I started trying to influence curriculum and structure, by providing all these resources that I felt that the mainstream faculty didn't have time to create. And, then, trying to get them to use these…Then, the other thing in a place like this (is) every course is offered in multiple sections, and I started working, trying to bring the sections into alignment. – Hattie, PhD SFES
We received an NSF grant to create a set of long-term projects, that will span multiple courses …The main goal is to embed students in something that's really very close to the process of actually doing scientific research, and from the very beginning. So they start these projects and enter biology from sort of the phenomenological point of view. Pick them up again in general physics to create some quantitative models. And, then pick them up again in some advanced biology courses, where they will elaborate them out into models of biological systems. And, so, from a curriculum development point of view, it's interesting. – Ron, PUI SFES
I've gotten a couple of curricular innovation grants for developing new courses. One of them is being taught by one of the faculty professors in the spring. I've not had a chance to teach it, but I developed the course…Classroom design was one of the big things. I designed the classroom that teaches 30 students now, and it's a lot more interactive and group focused, rather than individual students sitting at individual computers, doing their individual thing... Designing the distance education aspects, trying to get these courses all moved into a distance format that will do the same thing as the face-to-face class will do…so I think I've had quite a bit of influence...I talked to people about education and what kinds of things we could and should be doing, that have been backed up by research. – Teresa, PhD SFES
Well, we've had a large impact across the College of the Sciences. We definitely have, because we've done things like a National Science Foundation Course, Curriculum, and Laboratory Improvement grant on critical thinking…For two years, we took cohorts of 8 faculty across the College of the Sciences…that joined us for a week long summer institute to retool their class to teach for critical thinking ... So, so that's a big impact across the campus. – Cerra, PUI SFES
I think that when you have someone that's interested in curriculum and interested in instruction, I think it then motivates other faculty to take a look at how they are doing things in the classroom. One of the things we've just gone through recently was a review of our program and (in relation to) the MCAT. Most of our students are pre-professional students who want to be doctors, dentists, etc. We don't turn out a lot of science majors going on to graduate school…So, we sat down, and we looked at our curriculum, and we've had some impacts on the curriculum for our pre-professionals… And, we've revised our major, to have closer alignment with the standards of our nation. – Patty, PUI SFES
We're kind of the ones who push forward some of the curricular changes … they turn to us, figuring that we are at least much better versed in the literature and what has been done, and kind of where might be a logical place to pick up. So, in that respect, I think we're a resource for the department, that's perhaps a little bit different than somebody who is more classily trained and hasn't looked at education issues. Just like an organic chemist keeps a lot of the NMR spectrometers running. That's kind of their subspecialty … we keep the instruments that one would use in a classroom, kind of going and moving forward. – Opal, MS SFES
We took a course that has historically about a 50% success rate, and now has about a 75% success rate for first time through. We spent 18 months with three instructional specialists, an instructional designer, a leader of the center for teaching and learning, a multimedia specialist…redesigning it into a blended course … We are now doing it in (other courses) with remarkable success ... – Charlie, PUI SFES

Table G: SFES Impact on Undergraduate Science Education – Supporting Teaching Assistants (n = 13/50; 26%)

Sample evidence
I think the biggest influence I have is with the TAs. We have gone from having no TAs to now having seven or eight a year. And, many of them are right out of undergraduate and have never taught before. And, there's a huge difference. I mean, I can't remember the first time I went into a class… helping them with organization is, at the minimum, the thing that helps them be more effective teachers. And, you basically have their time they're being useful. They're supposed to get degrees and get out of here. – Mike, PUI SFES
I think the greatest impact I've probably had is with the graduate students within the department. I've worked for five years with grad students, teaching a graduate seminar course and I think that has had the greatest impact, in terms of how our teaching is viewed within the department, because these graduate students are kind of the front lines. They're out there teaching and planning what's going to be taught, and they'll go back and tell their faculty, you know, you should be doing this, you should be doing this, you should be doing this, within your classroom, and then they'll sort of seek me out and say, What do you do? I'm kind of curious about that, I want to know about that ... – Paula, PhD SFES
Well, we have TAs, and I work with them and get them interested in fulfilling learning objectives. I'm trying to give them some guidance and introduce them to some pedagogic concepts. And, so I'm influencing them. I'm not really influencing the other faculty in my department… the TAs are all undergraduates and they're only TAing freshman classes. So I work with them on the syllabus and timing things and review when they write the quizzes and tests…Some of the other professors just throw the lab notebook at a TA and walk away... – Gail, PUI SFES
I think (I've) had an impact, certainly on TA training. That we've got TAs who actually get some pedagogical training before we throw them in the classroom … – Rita, MS SFES
So, we added a recitation. And with that, we started training the TAs, because a lot of the TAs were taught traditionally, and we have a lot of also international TAs coming from places all over the world. We wanted to make sure we had some kind of fundamental training. So, we did a serious TA training that hadn't been done here in the past ... – Francine, PhD SFES
I converted all the labs to inquiry labs and trained all the grad students to teach those labs. Trained - I basically said, here's the labs, and this is the way we're doing it, Then we test to see if they are doing it the way we want them to do it. So, all the grad students are getting exposed to that kind of thinking. Then, I started this grad course, and in its third iteration, it's getting a lot of attention from others, from grad students in the program, and specifically, there are certain faculty who are very supportive of what we're doing. So, they recommend that the students take it, and there are other faculty that I never see any of their students. – Doug, PhD SFES
We started a TA training program that ended up spawning a whole letters and sciences TA training program… – Dina, PhD SFES

Table H: SFES Impact on Undergraduate Science Education – Contributing to Academic Assessment (n = 9/50; 18%)

**Sample evidence**
A lot of (my impact) has been with assessment. As the university has become more and more vested into documenting our effectiveness, our impact on our students to justify our existence, that's been a lot of what I have done…Everybody's sitting around going, "Oh, assessment, we can't possibly do assessment." You're kidding me, right? But, being able to envision what that would look like for a program, what it would look like for a major, or how you can pull together different pieces of information, I think has probably been what I first and foremost added to the department. – Opal, MS SFES
I transitioned, I was saddled with, I volunteered for – I don't know what the appropriate verb is – with the department assessment program. I got to be the Department Assessment Coordinator and that was probably a place where I had a lot of impact on the department, on the way we think about certain things. And I'm starting to see some fruit from that labor now. We're several years into a departmental assessment program that was not particularly welcome at the time … And, I was able to convince the people that were involved that if we were going to do this, we should do it well … So, we had learning outcomes, and I think I was influential early on in the mechanics of the assessment program, in explaining why this was important, and how we should do it. And, now, even the most diehard people who were against doing this, actually see the value of it … admitting now that these annual conversations are useful. <br>– Terrance, MS SFES
I think all of us have a big impact on our science departments through assessment …There's been a big push at our university, and all other universities, for more accountability and for us to have programmatic assessments in place. And, in order to have programmatic assessments, you have to have student learning outcomes for your courses. Our departments would not have gotten very far with that, or it wouldn't have been very sophisticated assessment, without the science education faculty. So, we definitely have helped in that arena … – Cerra, PUI SFES
We had to develop an assessment plan for our department. Trying to understand what it means to do outcomes assessment and to assess the learning of our students and to sort of set up learning targets and goals and outcomes, even just on that small of scale, that was pretty major ... I led that effort and tried to help people understand what that might look like ... Our department was charged with doing that, coming up with an outcome assessment document, and nobody really knew what that meant. So, in our department meetings, we hashed that out, and I think because I knew a little bit more about it, I helped lead that. – Wendy, MS SFES
We were in trouble with our regional accreditor … for not taking assessment seriously … And (the new administration) realized they needed people who had a history at the university who knew what had been going to move this forward. So, there are actually three of us faculty members. I have two colleagues who serve the same role, and they each serve two colleges, themselves …We had all been involved in assessment in our departments or colleges in some way or another, so we kind of took on the lead role for moving things forward, working with the new administration... – Lee, PhD SFES
I was Director of Evaluation and Assessment, and I've been a member of the Assessment Subcommittee of the Curriculum Committee for a number of years … So, when I was Director of Evaluation and Assessment that was the year that all the departments were required to write intended learning outcomes for their majors. And they were given guidelines: "Write three to five, and use them in a way that makes them measurable." And when we had 100% compliance on that, it was just astonishing to me! It's still astonishing to me to think about. Everybody did this… <br>– Theodora, PUI SFES

Table I: SFES Impact on Undergraduate Science Education – Fostering Involvement of Undergraduates in Research (n = 4/50; 8%)

Sample evidence
I started a program of mentored research for undergraduates at the freshman/sophomore level … a synergistic kind of relationship with the faculty on campus. So we'd mentor students out to labs to do a research project over the course of the semester. And, then, simultaneously, they would be enrolled in our second semester introductory lab. And, we required them to write about their research, doing a proposal about week five, and a final paper at week nine ...When I first started that, I got a lot of feedback from the faculty mentors because I was taking so much of my students' time away from the time that they could be in the lab by requiring them to write. Five years later, everybody was saying, "Keep them writing," because they discovered that the undergraduates were able to understand what they were doing and why they were doing it better than their graduate students, because of the writing requirement. – Dina, PhD SFES
One of the reasons they wanted to hire me was because I had been very strongly in favor of and involved in thinking about undergraduate research and research-based classes as great ways to learn science. And, because I had some evidence – at that time not a huge amount – about there being an attractiveness to both students and faculty of having this kind of approach instead of sit down/shut up learning … I was trying to have a chance to talk with all the different science departments about the importance of having students own their own findings and projects and finding the joy of discovery. – Annie, PUI SFES
The original goal was this: get the freshmen [discipline] majors in a room and introduce them on a very basic level to the research that the tenure track faculty are doing, because one of the things that has had the best improvement in STEM retention is getting students involved in research as early as possible. So, I had set the seminar up with that purpose in mind: get the students in the room, let the faculty come talk to them, and get them into research labs. – Tuan, PhD SFES
Well, actually, what I was interested in was looking at curriculum, looking how we can incorporate research into the curriculum and whether or not getting students interested in research early in their careers had an impact on whether or not they stayed in the sciences, past undergraduate work. What we have found is that, If you can involve them early in, that they actually in the laboratory. They prefer to have some open-ended questions and they'd rather not just have all your standard labs, that they think there's a value to both your take-them-out-of-the-lab manual labs, but they also appreciate and get excited about open-ended inquiry laboratories, especially if they see that they have an application in the real world… Some of the people I am working with are in the process of writing a chapter, and I am providing data for that chapter, which talks about how the culture has evolved at one of the institutions that was involved. I do know that in the collaboration, we had several publications … And, I know that the undergraduate research center concept has five models that have been -- in fact, I reviewed a book or an article that was published based on those five grants. So, I know the information is out there and that people have looked at those models to see how to incorporate undergraduate research into the experience for students. So, I guess there's probably a larger impact than what I'm aware of … – Patty, PUI SFES

Table J: SFES Impact on Undergraduate Science Education – Promoting Student Diversity and Retention (n = 4/50; 8%)

Sample evidence
We started a sort of zero-credit freshman seminar, just for our majors that we set up to figure out how to maintain our majors without losing half of them in the first year. So far, just having this zero-credit seminar – to get them all in the same room one day a week – seems to be providing some positive results. We don't lose nearly as many of our majors early in the process as we used to, and I think that's been a positive thing. – Tuan, PhD SFES
The dean is very excited to have the programs that we're doing, specifically the Learning Assistant Program, which is designed to improve retention, and our retention sucks. Apparently, he talks about it at the meetings of deans … All the department chairs on campus recently got a request from the president to say what we've done, what initiative we have to increased recruiting and retention. And, our new department chair put forth the Learning Assistant Program as our initiative, as something that makes a difference. It's getting some attention. Our DFW rate has dropped from 50% to 35% in the last year…which is not as good as I want it to be, but it's a pretty big improvement. – Tara, MS SFES
I was very much involved in encouraging my colleagues and working with them to get programs going for summer research for students from junior colleges who were students of color trying to think of transferring and wanting to have more encouragement ... – Annie, PUI SFES
Right now, the -- we have a grant from NSF that is aimed at recruitment and retention of underrepresented minorities and what can we do with students coming in to keep them in the pipeline ... – Phil, PhD SFES

3. Interview Protocol

Science Faculty with Education Specialties (SFES) Research Study

1. Preamble (5 minutes)

- Greetings, is this [interview subject's name]? Thanks so much for talking with us today, [interview subject]!
- There are two of us on the call today...My name is [interviewer 1 name], and I'm in the [science] department at [university name]...And I'm joined by my research colleague who will introduce her/himself... ([interviewer 2] introduces her/himself).
- As a reminder, we have two folks on the call today to make sure we understand as much as possible about your particular professional situation. So that we can go back and re-check our understanding, we'd like to record today's conversation, which would only be used internally and would never be shared publicly or associated with your name or institution. Can we turn the recorder on now?
- We sincerely appreciate your time, and we have no doubt that 1 hour will not be enough to learn all that we could learn from you and your experiences.
- The goal of this first interview is for you to share your story as a science education professional. In learning about your current position and your experiences, we hope to build a deeper understanding of what it means to be a science education professional, including the SFES phenomenon, and the term itself. We describe SFES positions as science department faculty positions – broadly including non-tenure track and lecture positions – that have roles in advancing science education. While we believe SFES is an appropriate description for your position, you may be less comfortable about that. Rest assured, there are no right or wrong answers. We value your views and want to understand your story and your perspective.
- While we have **7 main questions** we'd like to explore with you, we hope that our time together can be conversational and a chance for you to share your experience as a science education professional.
- After we have completed first interviews with all the participants in the study, we'll contact you to arrange a second, follow-up interview where we ask for clarifications, verify our interpretations, and give you a chance to share any additional information that has come to mind.
- Most importantly, everything you share with us will be kept strictly confidential. As fellow faculty supporting science education, we are highly aware of the complexities that come with these positions, and we will never share any information in publications or presentations of the study results that could link you with the data.
- You've already read and signed a copy of the Informed Consent document. Do you have any clarifying questions for us before we get started?
- Please feel free to ask for clarification at any time during the interview. So, are we ready to get started?

2. Current Position (10 minutes = 15 min to this point)

"Let's get warmed up by first talking about some of the basics of your current position."

What is your current academic position?

Probe as necessary:
- *department, rank, joint appointment*
- *tenure-track vs. non-tenure track, lecture vs. traditional faculty*

When did you start in this position?

Probe as necessary:
- *How long have you been at <name of institution>?*
- *Was this your original position at this institution?*

How did you get to this position?

Probe as necessary: What originally brought you here?

3. SFES Identity (10 minutes = 25 min to this point)

What is it about your position that makes it an SFES or SFES-like position?
How is your position and role as a science education specialist unique compared to other faculty positions in your department?

Recommended follow-up question: Although we describe SFES positions as science department faculty positions – broadly including non-tenure track and lecture positions – that have roles in advancing science education, how do you feel about this description?

Potential follow-up questions:

- Depending on their answer:
  - What are the essential activities that you do that make you an SFES? or
  - What essential activities are you NOT involved in that make your position NOT an SFES position?
- What do SFES faculty at your institution do that non-SFES faculty don't do?
- To what extent are you treated the same/differently than any other faculty member
  - Salary? Space? Access to grad students? RTP expectations?
- What professional societies, associations, etc. do you interact with in your role as SFES? What is the level and type of interactions you have? What are their strengths and limitations in terms of supporting you as an SFES?
- Do you see any benefits of a cross-disciplinary professional society for SFES? Why or why not?
- If you could give direct advice to national policy makers, such as the National Research Council, or funding agencies, what suggestions or changes in policy would you offer? Why?
- If someone were to ask you what is unique about SFES positions generally, how would you answer them? Why?

4. Motivations for the creation of SFES positions at this institution (5 minutes = 30 min to this point)

"Let's move to motivations for your position."

Why do you think your position was created?
Would others in your department or on your campus have different explanations?

Potential Follow-up Questions...

- Why do you think your position was created?
- What insights do you have about why you were selected, in particular, for this position?
- What is your sense of the goals for creating your SFES position?
- What is your sense of how varied the motivations were in creating your SFES position among campus stakeholders?
- Are there other SFES positions in your department? in other science departments? What is your sense of the motivations for creating those positions?
- If someone were to ask you why SFES positions have been increasingly created in science departments across the country, how would you answer them? Why?

5. Perceptions of the institutional impact and influence of SFES (5 min = 35 min to this point)

What impact or influence do you feel you have had in your position?
Probes: influence on your department? at your institution? in your discipline? in other communities?

Potential Follow-up Questions...

- ...beyond your department at the College level? University level?
- ...externally, on other institutions or groups outside your institution?
- How do you think others at your institution might describe your impact?
- Has your presence affected whether additional SFES hires are planned? Why or why not?
- If an administrator from another university asked you what the "added value" is of having an SFES in a science department, what might you tell them?
- If someone were to ask you what the potential impact would be of creating an SFES position in their department, how would you answer them? Why?

6. Information regarding training for SFES positions (5 min = 40 min to this point)

What professional training has best prepared you for your current position?

Potential Follow-up Questions...

- What aspects of your professional training have been most helpful to you in your work as an SFES? Why?
- What types of training do you wish you would have had that would most help you now in your SFES position? Why?
- How, if at all, has your professional training influenced your ability to do your work as an SFES? in obtaining funding for your work?
- If someone were to ask you what the most optimal training would be to prepare aspiring SFES, how would you answer them? Why?

7. Reasons for considering staying or leaving current position (10 min = 50 min to this point)

How satisfied are you in your current SFES position?
Have you seriously considered leaving it?
Recommended follow up and/or replacement for framing questions:

1. *What are the sources of satisfaction for you in your current position (i.e., why do you stay in your current position)?*
2. *What are the sources of dissatisfaction for you (i.e., what issues lead you to consider leaving)?*

- If Stayer: Why do you stay in your current position? What issues/changes/reasons would make you consider seriously leaving?
- If Leaver: What issues/reasons cause you to seriously consider leaving? What needs to change for you to change your mind?

Potential Follow-up Questions...

- Are you seriously considering staying in or leaving your current position? Institution? Field?
- What are your reasons?
- How similar/different do you feel your SFES experiences have been compared to other SFES that you have talked with? Why?

8. Other ideas about SFES that you would like to share (5 min = 55 min to this point)

What else about either you own SFES position and or the SFES phenomenon more generally would you like to share with us?

Potential Follow-ups...

- What else is important to know about your ideas about your SFES position or SFES positions more generally that we haven't talked about, yet?
- What else, if anything, would you like to share with us?

9. Next steps in the study (5 min = 60 min to this point)

- Do you have any concerns about what you have shared with us today?
- What's the best way to contact you for scheduling a 30-minute, follow-up phone interview in approximately 3 months?

10. Signoff (30 seconds = 60 min to this point)

"Thank you again for your time. We will be in contact about the follow-up interview, as well as the stipend paperwork. Enjoy the rest of your day/summer."
